# Change in COVID-19 outcomes in lymphoma patients across pandemic waves: results of the COVID-19-lymphoma registry of the Bavarian Cancer Research Center (BZKF) 2020–2022

**DOI:** 10.1007/s00277-026-07094-x

**Published:** 2026-08-31

**Authors:** Stefanie Forkl, Felix Freudenberger, Benedikt Jacobs, Sabine Einhell, Klaus Hirschbuehl, Rainer Claus, Dirk Hellwig, Dimitrios Mougiakakos, Martin Dreyling, Lena Illert, Simon Heidegger, Oliver Weigert, Johannes C. Hellmuth

**Affiliations:** 1 Department of Medicine III, LMU University Hospital, LMU Munich, Munich, Germany; 2 Bavarian Center for Cancer Research (BZKF), Lymphoma Study Group, Erlangen, Germany; 3 COVID-19 Registry of the LMU Munich (CORKUM), LMU University Hospital, LMU Munich, Munich, Germany; 4 Department of Medicine III, TUM School of Medicine and Health, TUM University Hospital, Technical University of Munich, Munich, Germany; 5 Department of Internal Medicine 5, Hematology and Clinical Oncology, Friedrich-Alexander-Universität Erlangen-Nürnberg, University Hospital Erlangen, Erlangen, Germany; 6 Department of Internal Medicine III, Hematology and Oncology, University Hospital Regensburg, Regensburg, Germany; 7 Hematology and Oncology, Medical Faculty, University of Augsburg, Augsburg, Germany; 8 Medical Faculty, University of Augsburg, Pathology, Augsburg, Germany; 9 Comprehensive Cancer Center Augsburg, Medical Faculty, University of Augsburg, Augsburg, Germany; 10 Department of Nuclear Medicine, University Hospital Regensburg, Regensburg, Germany; 11 Bavarian Center for Cancer Research (BZKF), Partner Site Regensburg, Regensburg, Germany; 12 Department of Hematology, Oncology, Cell- and Radiotherapy, Otto von Guericke University, Magdeburg, Germany; 13 Department of Hematology and Medical Oncology, University Medical Center Göttingen (UMG), Göttingen, Germany

**Keywords:** COVID-19, Lymphoma, Non-Hodgkin lymphoma, CD20-depleting therapies, Rituximab

## Abstract

Patients with malignant lymphomas are at increased risk for severe COVID-19. In the general population, outcomes of COVID-19 have improved dramatically over time due to increasing immunity through vaccination or infection and due to improved clinical care. It is unclear whether outcomes of COVID-19 in patients with lymphoma have improved similarly over time. We performed a multi-center retrospective study of lymphoma patients with COVID-19 at five tertiary care centers of the Bavarian Cancer Research Center (BZKF) in Germany, evaluating baseline characteristics as well as COVID-19 management and outcomes across different pandemic waves from March 2020 to March 2022. Clinical management of COVID-19 as well as vaccination rates changed significantly over time. In the most recent pandemic wave (cases after December 27th 2021), 88% of lymphoma patients had been vaccinated. Upon infection, 57% of patients were treated with monoclonal antibodies and 35% were treated with antiviral agents. Concurrent with these interventions, we observed markedly improved outcomes. Severe disease courses were significantly less frequent in the most recent pandemic wave compared to earlier pandemic waves (4/40 vs. 26/73, *p* = 0.03). Similarly, mortality was significantly lower in the most recent pandemic wave compared to earlier pandemic waves (2/40 vs. 14/73, *p* = 0.039). Although we cannot clearly distinguish the relative contribution of each intervention on the observed improvement in COVID-19 outcome, our data indicates that severity and mortality of COVID-19 in this vulnerable patient population has recently declined. Thus, our data largely supports a return to pre-pandemic treatment recommendations and protocols.

**Supplementary Information:**

The online version contains supplementary material available at https://doi.org/10.1007/s00277-026-07094-x.

## Main text

### Rationale

The clinical course of COVID-19 is highly variable, ranging from asymptomatic infections to acute respiratory distress syndrome (ARDS) and respiratory failure [1]. Patients with malignant lymphoma are at increased risk for severe COVID-19 with a mortality rate exceeding the general population [2, 3]. Immunosuppressive chemotherapy including B-cell depletion as well as immune deficiency associated with lymphoproliferative disease contribute to the severe and prolonged course of COVID-19 in lymphoma patients. Accordingly, consensus practice guidelines by EHA / ESMO advocated a tiered approach of delaying / omitting lymphoma treatment by magnitude of priority and clinical benefit [4].

Meanwhile, outcomes of COVID-19 in the general population have improved dramatically over time due to increasing immunity through vaccination or natural infection and due to improved clinical care including the use of monoclonal antibodies and antiviral therapeutics [5]. However, it is unclear whether outcomes of COVID-19 in lymphoma patients have improved similarly over time.

### Methods

We performed a multi-center retrospective study of lymphoma patients with COVID-19 at five tertiary care centers of the Bavarian Cancer Research Center (BZKF) in Germany. Inclusion criteria were active or past lymphoma disease and laboratory-confirmed diagnosis of SARS-CoV2 infection by antigen testing or reverse transcriptase polymerase chain reaction from respiratory material. Both inpatient and outpatient records were screened for eligible cases. Patients with a COVID-19 diagnosis between March 2020 and March 2022 were included. We distinguished five pandemic waves according to epidemiologic data collected at the Robert-Koch-Institute [6] (Supplementary methods). Data on baseline patient characteristics, lymphoma treatment, COVID-19 management and outcome were collected with a detailed case report form. Data cut-off was June 30, 2022.

### Results

Detailed clinical data on baseline characteristics, lymphoma treatment and outcome and COVID-19 treatment and outcome were available for *n* = 113 cases (Supplementary Table 1). Our cohort encompassed patients across 5 pandemic waves from January 2020 to March 2022.

Overall, median age of patients was 68 years (range: 23–92), 51% were male. The majority of lymphomas were low-grade B-cell Non-Hodgkin lymphoma (B-NHL, 45%) and high grade B-NHL (42%), followed by Hodgkin lymphoma (HL, 7.1%) and T-NHL (5.3%) (Supplementary Fig. 1).

The majority of the patients (86%) had received at least one lymphoma-directed therapy prior to COVID-19. Median time from last treatment to the timepoint of COVID-19 diagnosis was 33 days (range: 0–8758 days). 66 patients (58%) were treated within 6 months prior to COVID-19 diagnosis. Sixteen Patients were treatment naive (14%). Most patients (*n* = 78, 69%) had undergone CD20-depleting therapies (Rituximab or Obinutuzumab) and 59 patients (52%) had received a CD20-depleting therapy within 12 months prior to COVID-19 diagnosis. Details on types of prior treatment are given in Supplementary Table 1.

Lymphoma treatment was discontinued due to COVID-19 in 30 (27%) patients. At follow-up, 22 of these patients had resumed therapy with a median therapy break duration of 26 days (range: 5 to 90 days). Five patients deceased during therapy discontinuation with three of these patients having been assigned to best supportive care. Cause of death was COVID-19 in three cases and lymphoma as cause of death in one case (cause of death was unknown in one case). Progressive disease occurred during treatment discontinuation in 5 patients.

COVID-19 disease course varied widely across our cohort. 24 patients (21%) were completely asymptomatic while 89 patients (79%) reported symptoms consistent with COVID-19. 81 patients (72%) were hospitalized with 25 patients (22%) admitted to an intensive care unit for non-invasive ventilation (*n* = 14), invasive ventilation (*n* = 18) or extra-corporal membrane oxygenation (*n* = 4).

**Fig. 1:**
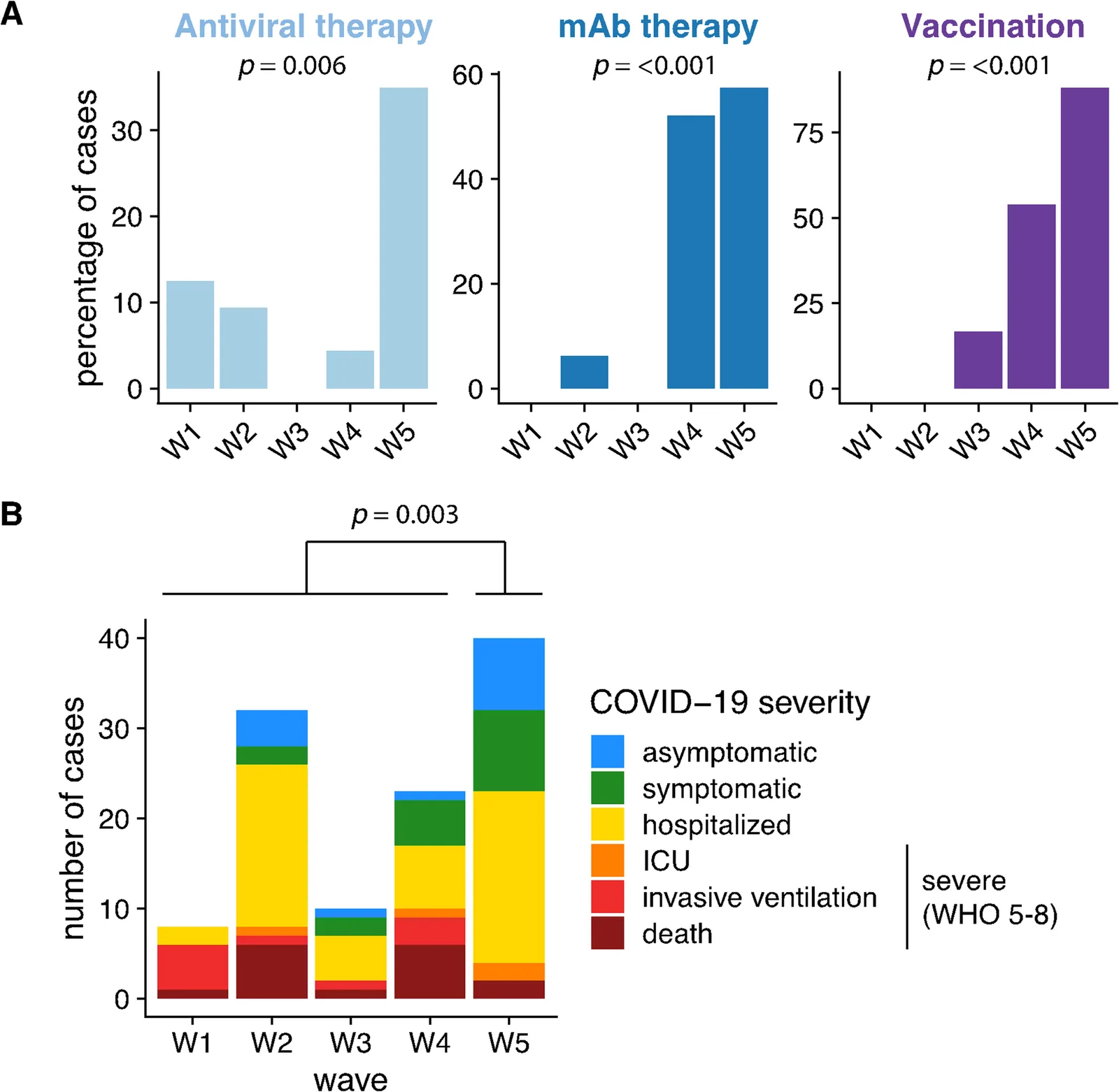
COVID-19 management and outcomes across pandemic waves. (**A**) Proportion of cases receiving antiviral therapy, monoclonal antibodies, or vaccination. (**B**) Distribution of COVID-19 severity grades, highlighting the number of severe cases (WHO ordinal scale 5–8). *p*-values calculated using Fisher’s exact test. mAb: monoclonal antibody therapy

Treatment was limited to best supportive care in 13 Patients (12%). The median age of these patients was 78 years (range 52–92 years), all had received at least one prior lymphoma-directed therapy and life expectancy related to lymphoma (i.e. irrespective of COVID-19) was estimated to be less than 12 months by the treating physician in 7 patients (53.8%). Clinical management of COVID-19 changed significantly over time. Monoclonal antibody treatment (namely, casirivimab/imdevimab and sotrovimab) became widely available during the fourth pandemic wave and was then widely administered to lymphoma patients in our cohort (12/23 and 23/40 patients in the fourth and fifth wave, respectively, Fig. 1A). Similarly, the use of antivirals was widely adopted in the fifth pandemic wave with 14/40 patients (35%) being treated with remdesivir (*n* = 8), molnupiravir (*n* = 5) or nirmatrelvir / ritonavir (*n* = 1).

Furthermore, vaccination rates increased with the availability of SARS-CoV2 vaccines reaching a rate of 88% in our cohort in the fifth pandemic wave (Fig. 1A).

Due to these substantial changes in management and vaccination rates, we asked whether COVID-19 outcome changed over time. Indeed, we observed a marked improvement starting with the fifth pandemic wave from December 2021. The duration of hospital admission was shorter during in the fifth pandemic wave compared to earlier pandemic waves (median 9.5 days [IQR 3.5–22.8] vs. 16.0 days [IQR 10.0–36.0]; *p* = 0.046; Supplementary Fig. 2) and severe disease courses were significantly less frequent (4/40 vs. 26/73, *p* = 0.003, Fig. 1B). Similarly, mortality was significantly lower in the fifth pandemic wave compared to earlier pandemic waves (2/40 vs. 14/73, *p* = 0.039, Fig. 1B). Of note, both cases that died due to COVID-19 in wave 5 had been assigned to best-supportive care resulting in a 0% mortality for patients without care limitation.

### Discussion

Our data indicates that the severity and mortality of COVID-19 in lymphoma patients has declined over time with a significant drop in the fifth pandemic wave starting in December 2021. Preceding and concurrent with this drop we observed substantial changes in SARS-CoV2 vaccination rates and COVID-19 management including the use of monoclonal antibodies and antiviral therapeutics. While we lack data SARS-CoV2 genotype in each individual case, omicron emerged as the dominant variant during the fifth pandemic wave in Germany and was associated with less severe COVID-19 disease course overall [7]. The main limitations of this study are its retrospective design, incomplete data for some variables, the relatively small sample size, and the marked clinical heterogeneity of the lymphoma cohort, which precluded meaningful multivariable and subtype-specific analyses. Consequently, we cannot clearly distinguish the relative contribution of vaccination- and/or infection-induced immunity, advances in COVID-19 management, and shifts in the SARS-CoV-2 variant landscape to the observed improvement in outcomes. Most likely, the observed improvement of COVID-19 outcome in lymphoma patients is caused by multiple factors including immunity after COVID-19 vaccinations and/or infection, advances in COVID-19 management and changes in the SARS-CoV2 variant landscape. Irrespective of the precise cause of improved COVID-19 outcome, our data largely supports a return to pre-pandemic treatment recommendations and protocols.

## Supplementary Information

Below is the link to the electronic supplementary material.

Supplementary Material 1

Supplementary Material 2

Supplementary Material 3

## Data Availability

anonymized data will be made available upon request.
