# Supplementary material for: Change in COVID-19 outcomes in lymphoma patients across pandemic waves: results of the COVID-19-lymphoma registry of the Bavarian Cancer Research Center (BZKF) 2020–2022
Source: Ann Hematol. 2026 Aug 31;105(9):401. doi: 10.1007/s00277-026-07094-x (PMC13526717; doi:10.1007/s00277-026-07094-x)
Supplement: Supplementary file 1 [file 277_2026_7094_MOESM2_ESM.pdf]

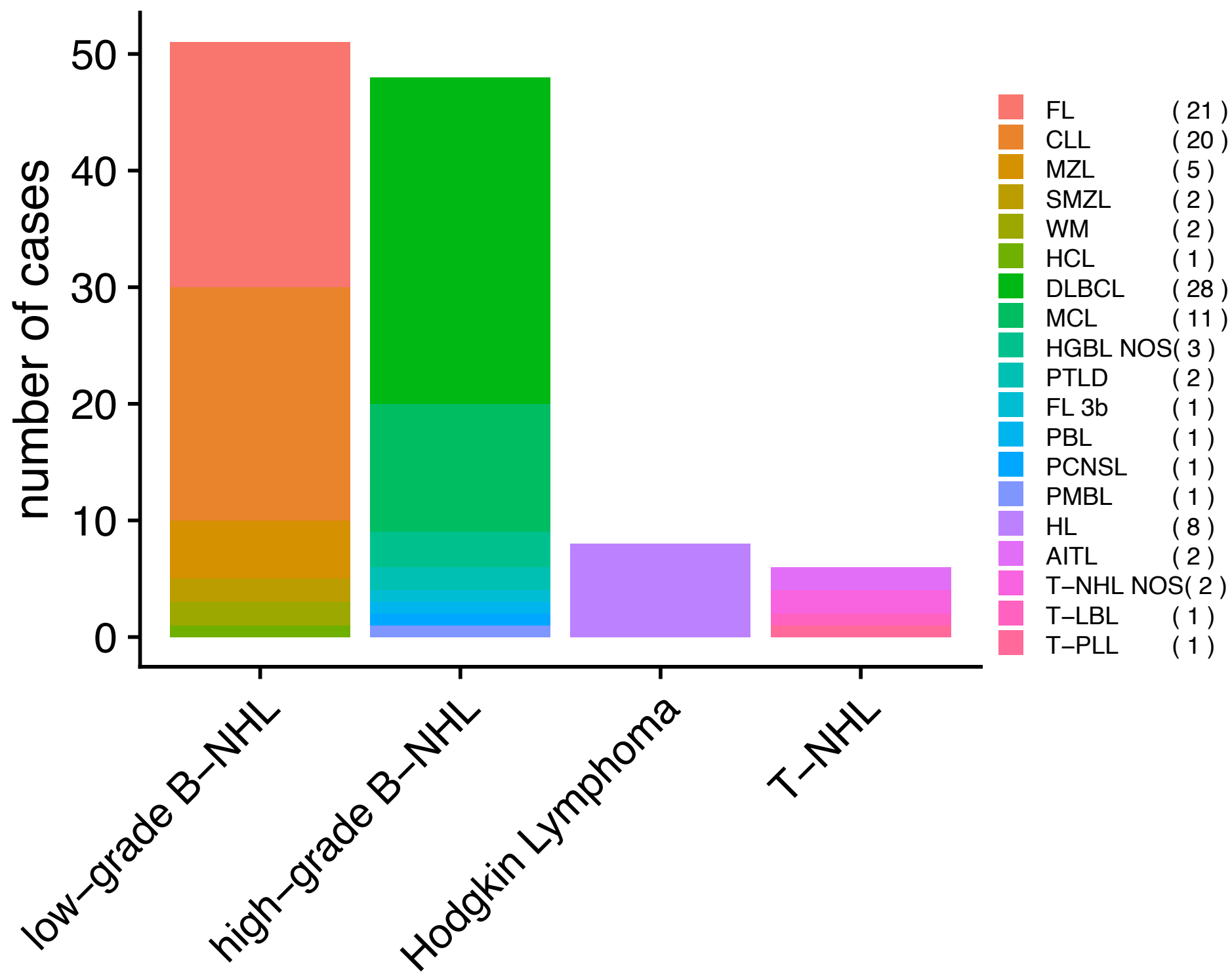

Supplementary Figure 1. Distribution of lymphoma subtypes in the study cohort. NHL: Non-Hodgkin lymphoma.

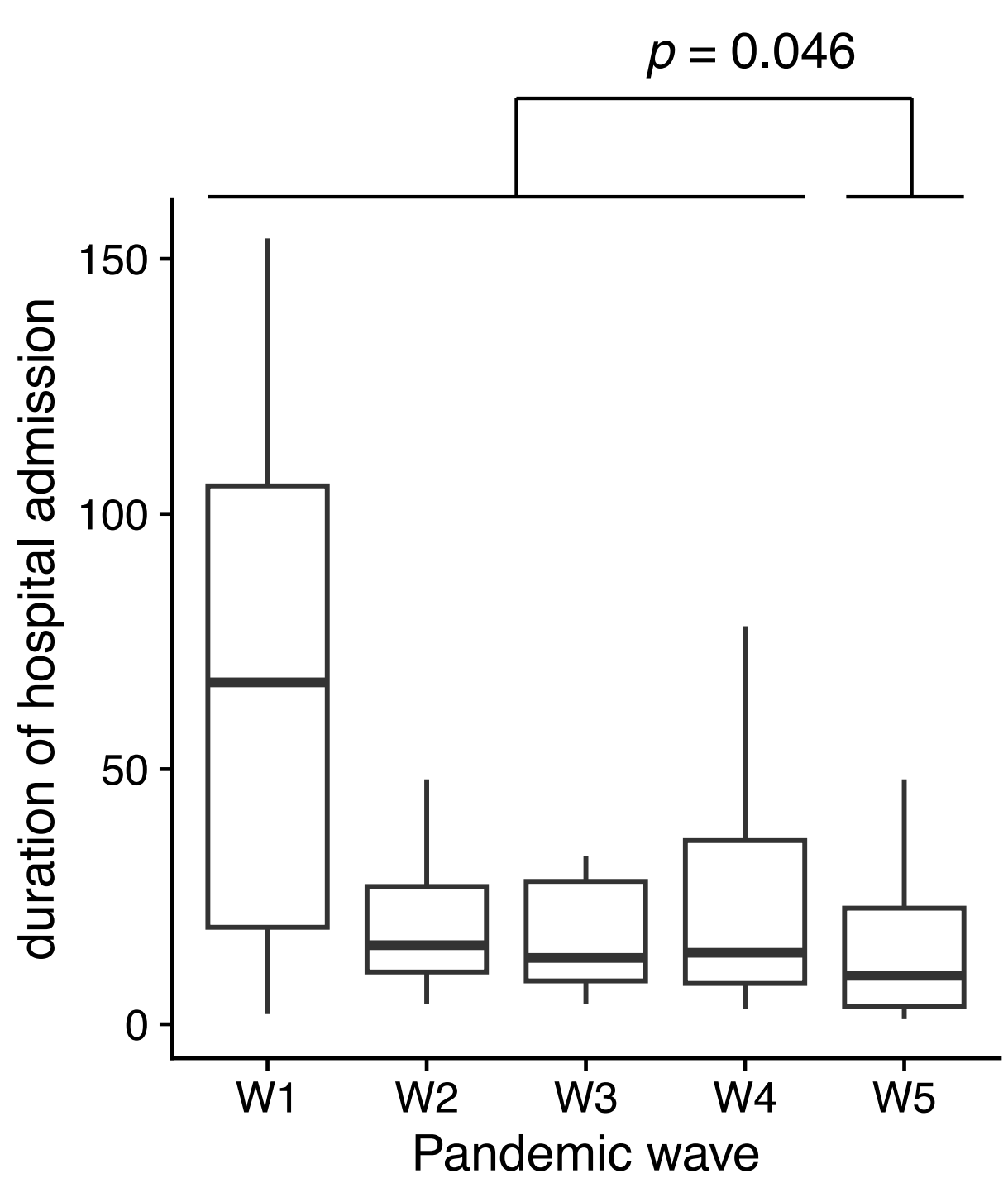

Supplementary Figure 2. Duration of hospital admission across pandemic waves. *p*-value calculated using Wilcoxon rank sum test.
