# Supplementary material for: Change in COVID-19 outcomes in lymphoma patients across pandemic waves: results of the COVID-19-lymphoma registry of the Bavarian Cancer Research Center (BZKF) 2020–2022
Source: Ann Hematol. 2026 Aug 31;105(9):401. doi: 10.1007/s00277-026-07094-x (PMC13526717; doi:10.1007/s00277-026-07094-x)
Supplement: Supplementary file 2 [file 277_2026_7094_MOESM3_ESM.pdf]

Supplementary Table 1. Baseline patient characteristics, lymphoma treatment, COVID-19 management and outcome in the study cohort.
IQR: interquartile range; SD: standard deviation.

**Patient Characteristics**	
Sex	
m	58 (51%)
w	55 (49%)
Age	
Median (IQR)	68 (57, 76)
Mean (SD)	65 (15)
Range	23, 92
Comorbidities	
Hypertension	53 (47%)
Smoker	8 (7.1%)
Diabetes	16 (14%)
COPD	9 (8.0%)
Cardiac disease	27 (24%)
Second cancer	10 (8.8%)
**Lymphoma Type and Disease Course**	
Lymphoma group	
low-grade B-NHL	51 (45%)
high-grade B-NHL	48 (42%)
HL	8 (7.1%)
T-NHL	6 (5.3%)
Number of therapies pre COVID	
0	16 (14%)
1	67 (59%)
>1	30 (27%)
Therapy status	
active	66 (58%)
post treatment	31 (27%)
treatment naive	16 (14%)
Lymphoma treatment	
Anti-CD20 monoclonal antibody	78 (69%)
Anti-CD20 monoclonal antibody within last 6 months	51 (45%)
Bendamustine	27 (24%)
Auto SCT	12 (11%)
Allo SCT	1 (0.9%)
CAR-T	7 (6.2%)
**COVID Treatment and Disease Course**	
Vaccination status	
vaccinated	23 (32%)
unvaccinated	50 (68%)
unknown	40
Time from latest Lymphoma treatment to COVID diagnosis [days]	
Median (IQR)	33 (12, 274)
Range	0, 8,758
Unknown	16
COVID Treatment	
Best supportive care	
yes	13 (93%)
no	1 (7.1%)
Unknown	99
mAb treatment	37 (33%)
Antiviral therapy	19 (17%)
COVID Outcome	
WHO score	
Asymptomatic (WHO 1)	14 (12%)
Ambulatory (WHO 2)	18 (16%)
Hospitalized (WHO 3-4)	51 (45%)
Hospitalized, severe disease (WHO 5-7)	14 (12,4%)
Death (WHO 8)	16 (14%)
Cause of death	
alive	85 (75%)
death due to COVID-19	16 (14%)
death due to lymphoma	9 (8.0%)
death due to other cause	3 (2.7%)
Duration of hospital admission	
Median (IQR)	13 (8, 34)
Mean (SD)	24 (27)
Range	1, 154
Unknown	34
