# Supplementary material for: Change in COVID-19 outcomes in lymphoma patients across pandemic waves: results of the COVID-19-lymphoma registry of the Bavarian Cancer Research Center (BZKF) 2020–2022
Source: Ann Hematol. 2026 Aug 31;105(9):401. doi: 10.1007/s00277-026-07094-x (PMC13526717; doi:10.1007/s00277-026-07094-x)
Supplement: Supplementary file 3 [file 277_2026_7094_MOESM1_ESM.docx]

**Supplementary Methods**

**Definitions**

**COVID-19 severity**: Covid-19 severity was classified according to the WHO ordinal scale ^1^:

| Patient state | Descriptor | Score |
| --- | --- | --- |
| Uninfected | no clinical or virological evidence of infection | 0 |
| Ambulatory | No limitations of activities | 1 |
|  | Activity limitation | 2 |
| Hospitalized, mild disease | Hospitalized, no oxygen therapy | 3 |
|  | Oxygen by mask or nasal prongs | 4 |
| Hospitalized, severe disease | Non-invasive ventilation or high-flow oxygen | 5 |
|  | Intubation and mechanical ventilation | 6 |
|  | Ventilation and additional organ support (pressors, RRT, ECMO) | 7 |
| Dead | Death | 8 |

**Pandemic waves**: We used the definition of the Robert-Koch-Institute to distinguish pandemic waves in Germany ^2^. The first pandemic wave was defined as the period from January 21, 2020 to September 27, 2020, the second wave from September 28, 2020 to February 28, 2021, the third wave from March 01, 2021 to August 1, 2021 , the fourth wave from August 2, 2021 to December 26, 2021, and the fifth wave from December 27, 2022 to end of inclusion date March 31, 2022.

**Treatment**: Patients in which time from last treatment to COVID-19 diagnosis was >6 months were classified as ‘post treatment’.

**Literature**

1	COVID-19 Therapeutic Trial Synopsis. https://www.who.int/publications-detail-redirect/covid-19-therapeutic-trial-synopsis (accessed May 7, 2024).

2	Epidemiologisches Bulletin 38/2022. https://www.rki.de/DE/Content/Infekt/EpidBull/Archiv/2022/38/Art_01.html (accessed May 7, 2024).
